# The use of an *in-vitro* batch fermentation (human colon) model for investigating mechanisms of TMA production from choline, l-carnitine and related precursors by the human gut microbiota

**DOI:** 10.1007/s00394-021-02572-6

**Published:** 2021-05-02

**Authors:** Priscilla Day-Walsh, Emad Shehata, Shikha Saha, George M. Savva, Barbora Nemeckova, Jasmine Speranza, Lee Kellingray, Arjan Narbad, Paul A. Kroon

**Affiliations:** 1 Quadram Institute Bioscience, Norwich Research Park, Norwich, NR4 7UQ UK; 2 Chemistry of Flavour and Aroma Dept, National Research Centre, 33 El Buhouth St, Giza, 12622 Dokki Egypt; 3 Foundation “Prof. Antonio Imbesi”, University of Messina, Piazza Pugliatti 1, 98122 Messina, Italy; 4 Department of Chemical, Biological, Pharmaceutical and Environmental Sciences, University of Messina, Polo Universitario dell’Annunziata, 98168 Messina, Italy

**Keywords:** Phosphatidylcholine, Lecithin, TMAO, Fish odour syndrome, Carnitine, Betaine, γ-Butyrobetaine, Cardiovascular disease, Metabolic disease, Human gut microbiota

## Abstract

**Purpose:**

Plasma trimethylamine-N-oxide (TMAO) levels have been shown to correlate with increased risk of metabolic diseases including cardiovascular diseases. TMAO exposure predominantly occurs as a consequence of gut microbiota-dependent trimethylamine (TMA) production from dietary substrates including choline, carnitine and betaine, which is then converted to TMAO in the liver. Reducing microbial TMA production is likely to be the most effective and sustainable approach to overcoming TMAO burden in humans. Current models for studying microbial TMA production have numerous weaknesses including the cost and length of human studies, differences in TMA(O) metabolism in animal models and the risk of failing to replicate multi-enzyme/multi-strain pathways when using isolated bacterial strains. The purpose of this research was to investigate TMA production from dietary precursors in an in-vitro model of the human colon.

**Methods:**

TMA production from choline, l-carnitine, betaine and γ-butyrobetaine was studied over 24–48 h using an *in-vitro* human colon model with metabolite quantification performed using LC–MS.

**Results:**

Choline was metabolised via the direct choline TMA-lyase route but not the indirect choline–betaine-TMA route, conversion of l-carnitine to TMA was slower than that of choline and involves the formation of the intermediate γ-BB, whereas the Rieske-type monooxygenase/reductase pathway for l-carnitine metabolism to TMA was negligible. The rate of TMA production from precursors was choline > carnitine > betaine > γ-BB. 3,3-Dimethyl-1-butanol (DMB) had no effect on the conversion of choline to TMA.

**Conclusion:**

The metabolic routes for microbial TMA production in the colon model are consistent with observations from human studies. Thus, this model is suitable for studying gut microbiota metabolism of TMA and for screening potential therapeutic targets that aim to attenuate TMA production by the gut microbiota.

**Trial registration number:**

NCT02653001 (http://www.clinicaltrials.gov), registered 12 Jan 2016.

**Supplementary Information:**

The online version contains supplementary material available at 10.1007/s00394-021-02572-6.

## Introduction

It is well established that the human gut microbiota produce substrates that are both beneficial and deleterious to health [1–3]. In particular, the metabolism of dietary substrates such as choline and l-carnitine to trimethylamine (TMA) and subsequently to trimethylamine oxide (TMAO) by a hepatic enzyme flavin-containing monooxygenase 3 (FMO3) is of interest due to the strong associations of high circulating TMAO concentrations with the risk of numerous metabolic diseases and the risk of death from heart failure [4–6]. Whether or not TMAO is causally linked to disease risk remains contentious, but there is a growing consensus that changes in microbial production of TMA are indicative of alterations in gut microbiota composition and diversity [7–10], i.e. that high levels of circulatory TMAO reflect gut dysbiosis in some way. In addition, because they are essential for methylation processes, lipid membrane generation, lipid metabolism and neurotransmitter production, excessive metabolism of choline and l-carnitine by the gut microbiota may deprive the host of these essential substrates and increase the risk of metabolic diseases such as diabetes, cardiovascular diseases and neurodegenerative diseases [11]. Accordingly, there is considerable interest in developing effective and sustainable interventions that effectively reduce TMA production, and, therefore, host TMAO burden.

The requirement for the gut microbiota in the metabolism of choline to TMA was elegantly demonstrated by Tang et al. who showed that the metabolism of phosphatidylcholine (PC)/lecithin to TMA, and subsequently TMAO, could be suppressed by the administration of broad-spectrum antibiotics, without suppressing the production of choline and betaine [10]. Metagenomic and transcriptomics approaches have played a crucial role in identifying genes, enzymes and pathways that are involved in TMA production [12, 13]. However, the presence of genes in microbial genomes that are predicted to be capable of degrading choline and l-carnitine is not always indicative of functional capacity of the microbe to produce TMA [14, 15]. Consequently, animal, human and *in-vitro* single-strain studies have been employed to functionally characterise the pathways and species involved in the metabolism of choline, l-carnitine, betaine and γ-BB. Of note are the requirement of a glycyl radical enzyme choline TMA-lyase (CutC) along with its activating enzyme (CutD) for the metabolism of choline to TMA, [15–17]. The importance of bacterial microcompartments in choline metabolism has also been demonstrated using bacterial pure cultures [18]. In mice, using PC as a substrate, it was reported that TMA can be formed as a result of multiple steps (PC → choline → betaine → TMA), although betaine was a poor substrate for the production of TMAO [19, 20]. Another study incorporating experiments with single bacterial strains, live human faecal microbes and mouse caecal cell lysates provided evidence that the metabolism of choline to TMA could be inhibited by a choline analogue 3,3-dimethyl-1-butanol (DMB) [21]. Nevertheless, in humans it is still unclear which pathways are predominant for the metabolism of choline to TMA. The absolute requirement of the gut microbiota in the metabolism of l-carnitine to TMA has also been demonstrated, in single-strain, animal and human studies [22, 23]. Using a combination of bioinformatics and strain-specific approaches, it was shown that the Rieske-type two component l-carnitine oxygenase/reductases (CntA/Bs) are required for microbial dependent degradation of l-carnitine to TMA [14]. In another report, it was shown that the concentration of TMAO in plasma peaked 24 h (h) after oral l-carnitine administration [22]. Using both human and animal studies, Koeth et al.provided evidence that γ-BB is an obligate intermediate formed during the metabolism of l-carnitine to TMA with the major site for γ-BB production being the small bowel, proximal to that of TMA production [22].

Here we describe the use of an *in-vitro* batch fermentation model to study the metabolic pathways for TMA production from dietary substrates such as choline, l-carnitine, betaine and γ-BB in humans. This model has been widely used to investigate the microbial metabolism of various other nutrients [24, 25]. We hypothesised that the conversion of these substrates into TMA in a batch model faithfully replicate the findings from previously reported studies of TMA production in human, animal and in-vitro studies. In testing the human colon model for this purpose, we anticipate being able to study the human gut microbiota-dependent conversion of these substrates to TMA in more detail, and demonstrate that this model is suitable for studies that seek to find dietary interventions that reduce the production of TMA. Our specific objective was to measure the changes in the concentration of TMA and associated metabolites over an extended period (24 h) in the colon model when incubated with TMA precursors. We also explored whether DMB, which had previously been shown to inhibit TMA production from choline in-vivo and in single-strain models [21], would inhibit the metabolism of choline to TMA in the human *in-vitro* colon model. In addition, we investigate whether the conversion of these substrates to TMA could be replicated using a simple anaerobic environment without pH control.

## Materials and methods

### Materials

Trichloroacetic acid (TCA), heptafluorobutyric acid (HFBA), γ-BB (3-(carboxypropyl)trimethylammonium chloride) and choline chloride-(trimethyl-d9) were purchased from Sigma–Aldrich. All unlabelled methylamines except γ-BB, all solvents unless otherwise stated, acetic acid, and ammonium acetate were purchased from Fisher Scientific Limited. Labelled internal standards [(d9-TMA), d9-TMAO and d9-carnitine] were purchased from Cambridge Isotope Laboratories. Fermac 260 pH control units were from Electrolab, and Stomacher 400 from Seward. 3,3-Dimethyl-1-butanol (DMB) was purchased from VWR^™^.

### Study design

An *in-vitro* batch fermentation model was used to study the microbial metabolism of choline, betaine, l-carnitine and γ-BB. Faecal samples were obtained from participants recruited onto the QIB Colon Model study. Men and women aged 18 years or older who live/work within 10 miles of the Norwich Research Park were recruited if they satisfied the following criteria: participants who were assessed to have a normal bowel habit, regular defecation between three times a day and three times a week, with an average stool type of 3–5 on the Bristol Stool Chart, and no diagnosed chronic gastrointestinal health problems, such as irritable bowel syndrome, inflammatory bowel disease, or coeliac disease. Participants were asked further questions immediately prior to donating a stool sample to confirm that they had not taken antibiotics or probiotics within the last 4 weeks, had not experienced a gastrointestinal complaint, such as vomiting or diarrhoea, within the last 72 h, were not currently pregnant or breast-feeding, and had not recently had an operation requiring general anaesthetic. The study was approved by the Quadram Institute Bioscience (formally Institute of Food Research) Human Research Governance committee (IFR01/2015), and London—Westminster Research Ethics Committee (15/LO/2169). The informed consent of all participating subjects was obtained, and the trial is registered at http://www.clinicaltrials.gov (NCT02653001). Twenty-one fresh human faecal slurry from five different donors were used for choline inoculations. For l-carnitine, betaine and γ-BB inoculations, samples from three donors were used in eight, four and six fermentation experiments, respectively, with each donor being used at least once for each substrate.

Each substrate was added to vessels to give a target concentration of 2000 µmol/L. A blank vessel was inoculated with PBS and human faecal slurry. Concentrations of TMA and the related precursors were measured at baseline, then at 2, 4, 6, 8, 10, 12 and 24 h. In three subsequent experiments, 2000 or 10,000 µmol/L DMB was added to vessels treated with choline. Finally, similar substrate concentrations for choline, l-carnitine, betaine, γ-BB were also used for the anaerobic fermentations without pH control.

### In-vitro batch fermentations

Batch fermentation *in-vitro* human colon model experiments were carried out as previously described by Parmanand et al. and depicted in supplementary Fig. 1 [24]. All vessels were assembled, sealed and autoclaved before each experiment. Twenty-four h prior to experiments, colon model media (2 g/L each of peptone water, yeast extract and NaHCO3, 0.1 g NaCl, 0.04 g/L each of K_2_HPO_4_ and KH_2_PO_4_, 0.01 g each of MgSO_4_.7H_2_O, CaCl_2_.6H_2_O, 2 ml Tween, 10 g/L d-glucose, 10 µl vitamin K, O.5 g/L each of cysteine and bile salts) was added into vessels and continuously supplied with oxygen free nitrogen with continuous stirring throughout the experiment as described by Parmanand et al. [24]. On the day of treatments, a fresh human faecal sample was diluted 1/10 w/v with phosphate buffer and homogenised using a Stomacher 400 for 45 s at 230 RPM to generate the faecal slurries that were used as inocula. Master stocks of 1 M of each of l-carnitine (99 + %), choline chloride (99%), betaine hydrochloride (99% extra pure) and γ-BB (technical grade) were prepared in PBS and filter sterilised using 0.22 µm Minisart filters. To achieve a final concentration of 2000 µmol/L, 220 µl of the substrate stocks were added separately to vessels containing faecal slurry (final concentration of 1 g stool per 100 mL colon model contents) and made up to a final volume of 110 ml with growth media. Based on a published average of the number of microorganisms in 1 g of wet stool [25], the 1.1 g of stool used in each fermentation vessel is expected to contain an average of 0.96 × 10^11^ microorganisms. In selected experiments using choline as substrate, DMB was also added to a final concentration of either 2000 or 10,000 µmol/L to estimate the extent to which DMB was able to inhibit TMA formation from choline. pH was maintained at 6.6–7.2 using Fermac 260 pH control units and pumps that automatically added either 1 M HCL or 1 M NaOH when the pH was out of this range. The vessels were maintained at 37 °C using a circulating water jacket. Samples were collected every two hours for 12 h and then at 20 h and 24 h and frozen immediately at − 20 °C. It is worth noting that while each substrate stock was generated and added to give an expected final concentration of 2000 µmol/L, the concentrations of some substrates measured at the beginning of experiments were materially lower than 2000 µmol/L. It was possible that this was due to complexing of the substrates with faecal components (e.g., fibre/undigested food) such that they were lost in the pellet after the centrifugation step to clarify samples before analysis. Quantification of the substrates in PBS alone gave concentration estimates for betaine and l-carnitine that were approximately 2000 µmol/L, and 1611 and 1685 mol/L for γ-BB and choline, respectively. It was shown that the choline was contaminated with TMA, and that the sum of choline plus TMA gave a total concentration of approximately 2000 µmol/L. The γ-BB used was technical grade, and although it was not contaminated with TMA, it was presumably contaminated with one or more unknown substances. This is further clarified in Table 1 where the concentrations listed in column 4 (‘Measured in PBS’) are the best estimates of the total concentration of each substrate in the experiments and the concentrations listed in column 3 (‘Measured in 1% faecal matrix’) likely best reflect the available (in solution) concentrations.

**Table 1:** Analysis of TMA substrates and TMA in the complete colon model matrix and in PBS

Substrate	Theoretical conc^n^ (µmol/L)	Measured in 1% faecal matrix (µmol/L)	Measured in PBS (µmol/L)	Measured TMA conc^n^ (µmol/L)	Sum of substrate _(PBS)_ + TMA (µmol/L)
Blank	0	n.d	n.d	n.d	0
Choline	2000	1742	1685	234	1919
Betaine	2000	1883	1936	n.d	1936
l-Carnitine	2000	1658	2087	n.d	2087
γ-BB	2000	1536	1611	n.d	1611

The concentrations of the substrates that had been supplemented to achieve a 2000 µmol/L concentration in a 1% faecal slurry in colon model media (colon model starting conditions) were lower than expected (column 3). This was not due to the matrix affecting the quantification as standard curves were matrix-matched. Since this effect may have been due to binding of substrates to matrix components (e.g., fibre from the faecal sample), we also measured the substrate concentrations in PBS which gave modestly higher estimates for betaine and γ-BB and a significantly higher estimate for l-carnitine (2087 versus 1658 mol/L; column 4). A significant concentration of TMA was observed in the choline supplemented samples but not in any of the samples supplemented with the other substrates (column 5); this was a consistent observation across different batches of choline. Together, these observations show that (i) the supplementations with betaine and l-carnitine were within 4% of the target concentration of 2000 µmol/L, (ii) that a significant proportion (~ 20%) of the l-carnitine was not in the aqueous phase and may not be available for metabolism by the gut microbiota, (iii) that supplementations with choline achieved concentrations of about 1750 µmol/L and were contaminated with TMA that roughly accounted for the ‘missing’ choline, and (iv) γ-BB supplementations only achieved concentrations around 1600 µmol/L, presumably due to contamination of the purchased γ-BB material

### Metabolite quantification using LC–MS/MS

Serial dilutions of all metabolites were prepared in 1% filtered faecal slurry to match the matrix of the samples for the calibration curves. Prior to analysis, samples were filtered and 5 µl of sample was mixed with 25 µl of 50% trichloroacetic acid (TCA) and kept at 4 °C for 5 min for deproteinisation. 70 µl of isotopically labelled internal standards (d9-choline, d9-TMA, d9-TMAO and d9-carnitine) prepared in 0.2 M acetic acid were added to the samples and the serially diluted standards, and microcentrifuged at maximum speed for 5 min. 5 µl of the mixture was transferred to chromatography vials containing 95 µl milliQ water for analysis with LC–MS/MS (Agilent Technologies, USA). Briefly, Mobile phase A contained 10 mM ammonium acetate and 0.05% heptafluorobutyric acid (HFBA) in water while mobile phase B consisted of 10 mM ammonium acetate, 0.05% HFBA in 90% methanol. The gradient was started with 0.2 ml/min flow from 2% mobile phase B which was increased by 10% within 1.54 min and after washing for 4 min, equilibration was for another 2.5 min. The total run was 8 min. The separation was carried out on Waters C8 (100 X2.1 mm, 1.7 µm) column and temperature was kept at 35 °C. The 6490 MS/MS system equipped with an electrospray ionization (ESI) source operated in positive-ion detection mode was used. Nebulisation, disolvation and collision was carried out using nitrogen gas and multiple-reaction monitoring (MRMs) mode was used with an Agilent optimizer software to optimise ion and energy collision. A gas flow of 12 L/min with gas temperature of 200 °C were used along with the temperature of 300 °C and flow rate of 11 L/min for sheath gas. 50 psi, 3500, 1000 V, were used for nebuliser pressure, capillary voltage and nozzle voltage, respectively. The iFunnel radio frequencies (RF) were 150 V RF for high pressure and 60 V for low pressure RF. The flow of LC eluent was sprayed into MS interface without splitting. Retention times and MRM transition (precursor/product) ions were used to identify and quantify the metabolites (Table 2). Sample peak area/isotopically labelled internal standards peak area ratios were used to calculate concentrations using standard curves.

**Table 2:** Retention times and target ion masses for metabolite identification and quantification using LC–MS/MS

Compound	Retention time (min)	Precursor ion (m/z)	Product ion (m/z)
Betaine	0.84	118.2	58.1
l-Carnitine	0.95	162.2	43.1
D9-l-Carnitine	0.93	171.2	43.1
Choline	1.59	104.2	45.1
D9-Choline	1.59	113.8	49.2
TMA	1.70	60.6	44.1
D9-TMA	1.68	69.4	49.2
TMAO	1.43	76.1	58.1
D9-TMAO	1.45	85.2	66.1
γ-BB	1.12	146.2	86.9

### Statistical analysis

To assess whether choline, betaine, l-carnitine and γ-BB are at all metabolised to TMA in the multi-species batch fermentation human *in-vitro* colon model, we compared the increase in the concentrations of TMA over 24 h to the increase in the untreated ‘Blank’ fermentations. To account for within-sample correlation in outcomes, and the observation that standard deviation of TMA concentration was proportional to the mean values in each condition, a linear weighted mixed model was used to estimate the differences between production from different substrates, with regression weights set to a power of the residual variance. A random effect of substrate for groups defined by sample was included in the model. The average marginal effect of adding each substrate on TMA produced was estimated, as well as all pairwise differences between substrates. The packages nlme (version 3.1–139) and emmeans (version 1.3.4) in R statistical software (version 3.6.1) were used to perform the statistical analysis [26–28]. All trajectories of metabolite levels over time periods under each treatment condition, and the difference in choline metabolism without DMB or with 2 or 10 mM/L DMB are described graphically, both as means within treatment groups and for each fermentation individually.

## Results

### Significant production of TMA from choline, l-carnitine and γ-BB but limited production from betaine

**Fig. 1:**
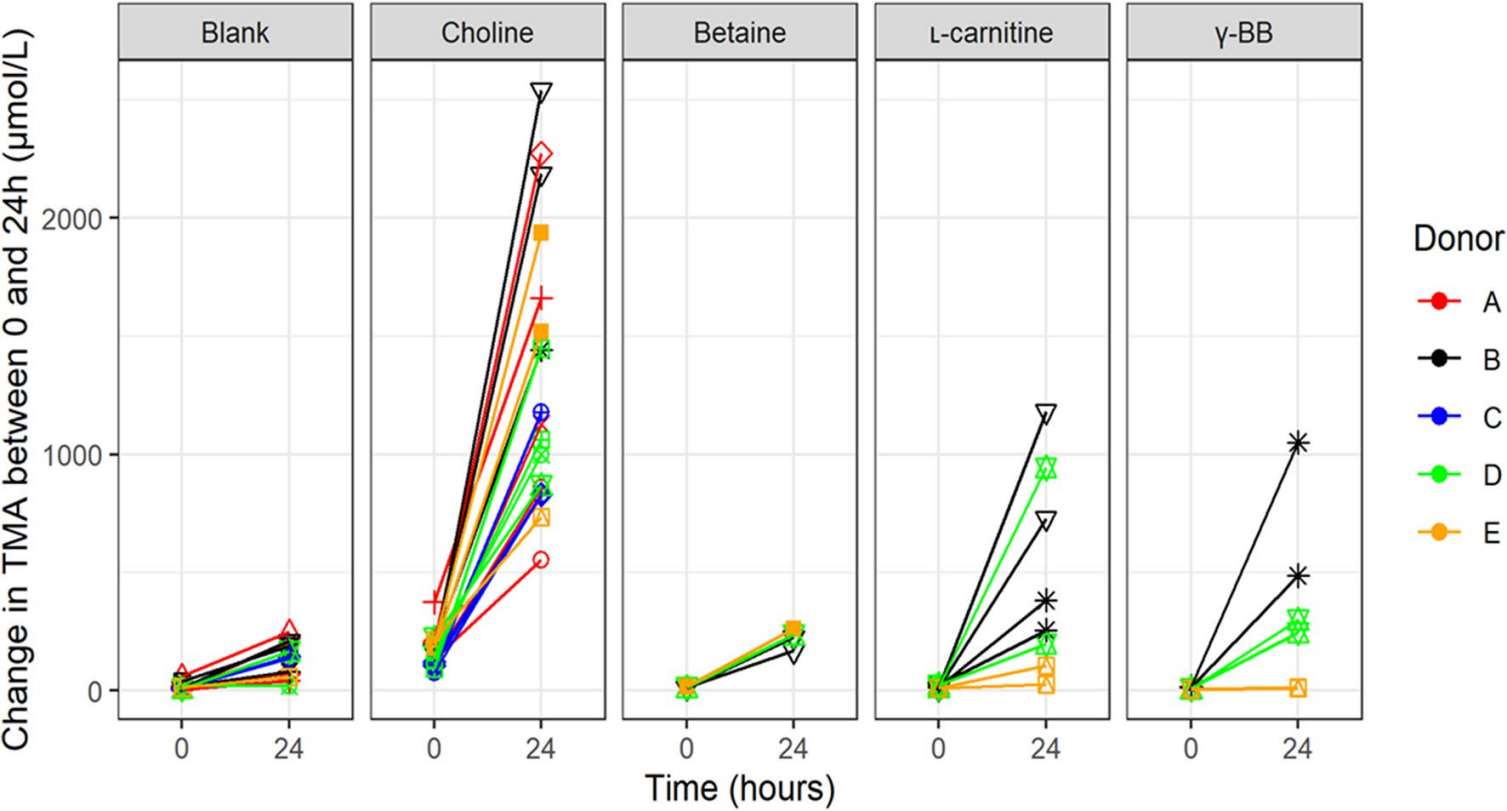
Change in TMA between 0 and 24 h in each batch fermentation seeded with faecal samples of five different donors. Fermentation stratified by substrate added. Colours correspond to donors and marker shapes to individual experiments. There is a high intra-class correlation between replicate fermentations within experiments, but little intra-class correlation within donors beyond this. Variation in the levels of TMA produced across experiments is high for each substrate, despite similar levels of substrate being utilised

The change in TMA concentration after incubation of the human faecal inoculum with each substrate was assessed after 24 h, and the data are presented in Fig. 1 and Supplementary Table I. Differences in production between substrates with confidence intervals are shown in Supplementary Table 1. Average TMA increased between 0 and 24 h for all treatments, including the blank. Large variations were observed in TMA production in different experiments (Supplementary Fig. 2, 3).

**Fig. 2:**
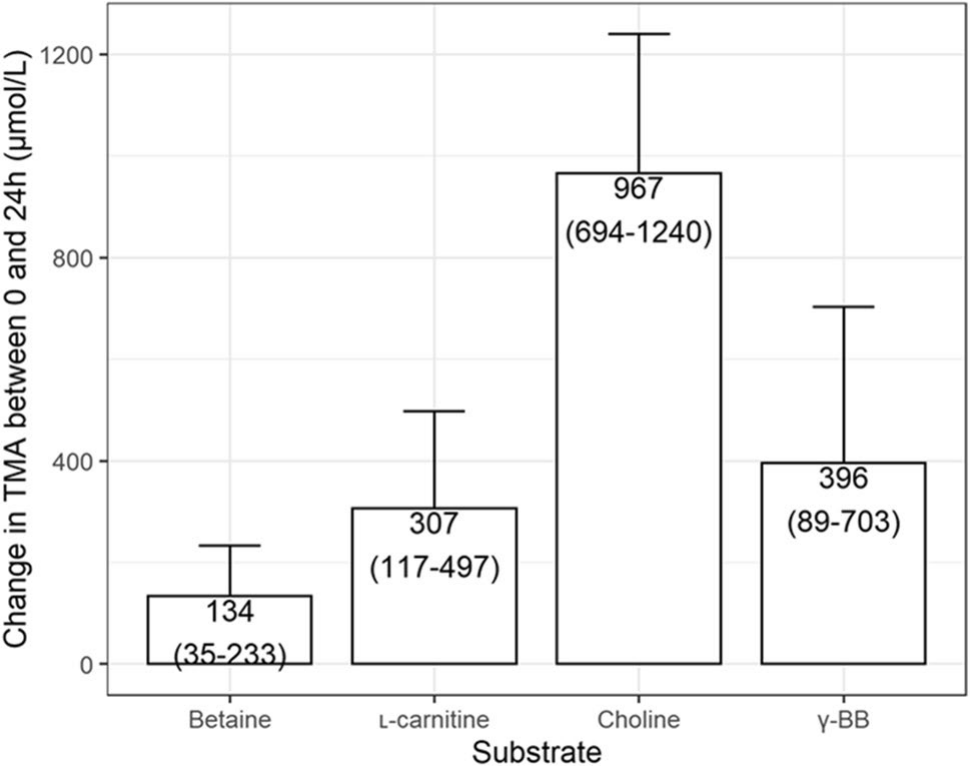
Estimated mean TMA produced from each substrate over 24 h. Estimates were obtained by mixed effects regression models of the difference between TMA concentration at 0 and 24 h (as described in the statistical methods). The effect of each substrate is calculated with correction for the production of TMA observed in blank vessel without added substrates. Error bars represent 95% confidence intervals

TMA produced after 24 h from choline is consistently greater and was on average significantly higher than that produced from other substrates (Fig. 2 and Supplementary Table 1). TMA produced from betaine was low in all cases. At 24 h, TMA from l-carnitine and γ-BB was highly variable across experiments, ranging between 0 and 1000 µmol/L, with no differences in average production between the two (Fig. 2 and Supplementary Table 1).

### Metabolic pathways for individual substrates

**Fig. 3:**
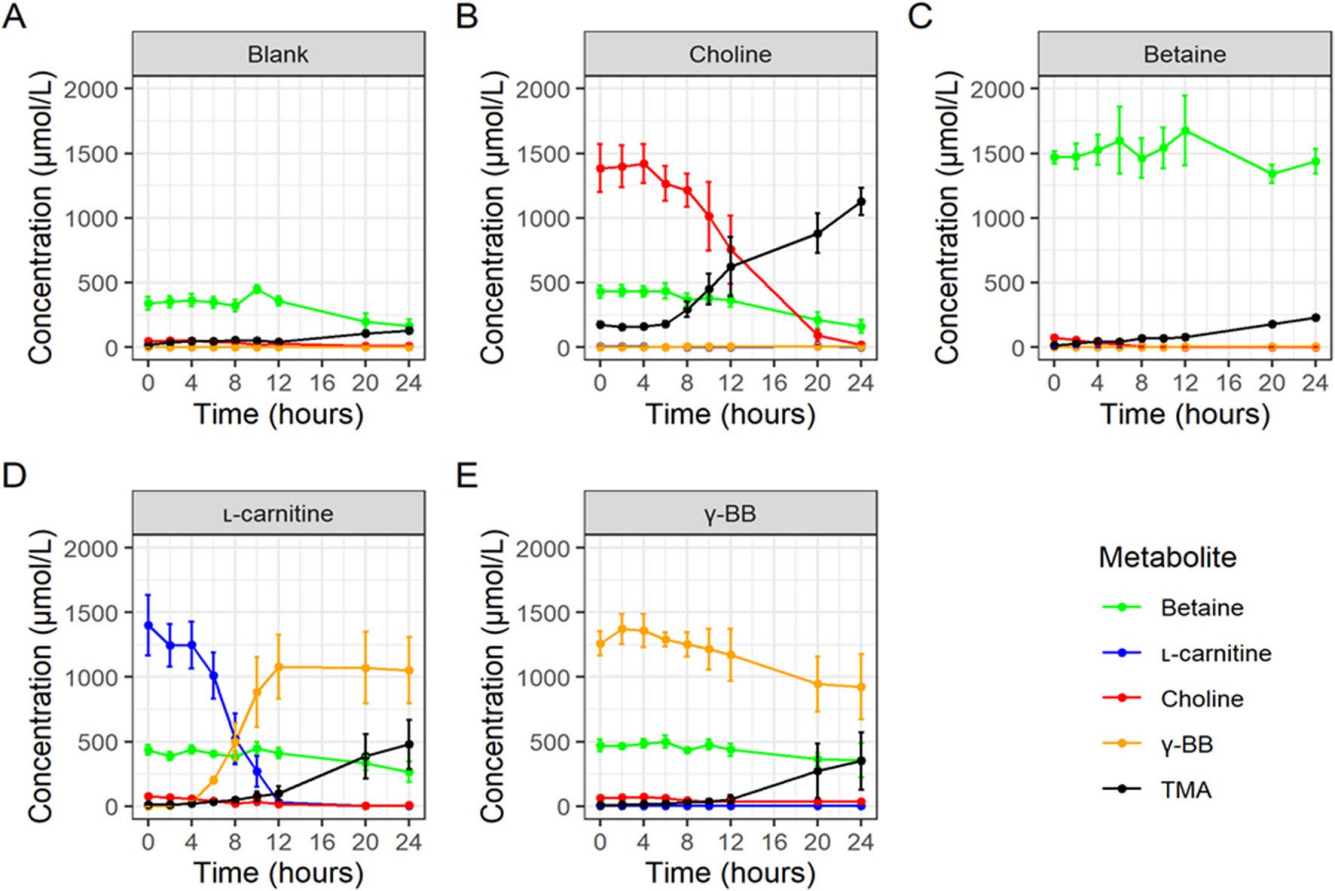
The average trajectory of all metabolites. With no added substrate (**a**) and following supplementation with each substrate (**b**–**e**). Error bars represent standard errors

The trajectories for substrate metabolism and TMA production varied between treatments. Figure 3 shows the average trajectory of each measured metabolite over time under each treatment condition. Trajectories in individual fermentations are shown in Supplementary Fig. 4. In the blank, an increase in TMA was observed at 20 and 24 h (Fig. 3a).

### The direct choline TMA-lyase pathway is the predominant route of colonic TMA production from choline with little or no contribution from the betaine aldehyde-choline dehydrogenase-betaine reductase pathway

In the choline inoculated fermentations, the concentration of choline started to decline between 6 and 8 h and continued to do so until 24 h by which time it was almost completely utilised in all experiments (Fig. 3b). TMA levels started to increase from the baseline concentration between 6 and 8 h which corresponded with the decrease in baseline levels of choline. It is possible that betaine could be an intermediate in the metabolism of choline to TMA which in theory can occur via the aldehyde/choline dehydrogenase /betaine reductase pathway. However, we did not observe a significant decrease in betaine values when it was used as the substrate (Fig. 3c). However, we did observe modest increases in the concentrations of TMA in betaine-supplemented incubations, and although some of this could be accounted for by reductions in the concentrations of choline and other substrates present in the baseline samples, the final concentration of TMA was significantly higher that observed in the blank incubations, suggesting that some of this TMA may have come from the metabolism of betaine. Additional evidence that there was at least a slow production of TMA from betaine was the observation that background betaine concentrations declined slowly across 24 h in all the non-betaine-supplemented incubations (Fig. 3a, b, d, e). It is also interesting to note that the absolute concentration of betaine in baseline samples, which originates from the faecal inoculum, was substantial (almost 500 µmol/L), which is consistent with a very slow rate of conversion of betaine to TMA and, therefore, the accumulation of unmetabolized betaine in the human colon. Overall, and taken together with the observation here that appearance of TMA closely mirrored the disappearance of choline, the data presented do not support the notion that the indirect pathway via betaine is a significant route of TMA production from choline by human gut microbiota.

### The metabolism of l-carnitine to TMA requires the γ-butyrobetainyl-CoA:carnitine CoA transferase-carnitine TMA-lyase pathway with formation of γ-BB as an intermediate and does not occur via the Rieske-type monooxygenase or the l-carnitine dehydrogenase pathway

In the l-carnitine supplemented fermentations, the concentration of l-carnitine declined steadily from 6 h until 12 h, by which time it was no longer detectable (Fig. 3d), whilst only a minimal increase in the concentration of TMA was observed during this time. However, there were substantial increases in γ-BB concentrations up to and peaking at 12 h, and these closely corresponded with the disappearance of l-carnitine. Taken together, the observations that (i) the choline observed at baseline also declined from 6 h, (ii) at no timepoint did the concentration of betaine increase and (iii) the concentration of betaine was lower at 24 h than at 0 h, all support the notion that little if any betaine is formed from l-carnitine by the human gut microbiota (Supplementary Fig. 4). Therefore, the production of TMA from carnitine likely occurs via the γ-butyrobetainyl-CoA:carnitine CoA transferase-carnitine TMA-lyase pathway with the formation of γ-BB as an intermediate.

### y-BB is metabolised to TMA but not to l-carnitine, and at a slower rate than the conversion of choline to TMA

In the γ-BB-supplemented ermentations, the concentration of γ-BB declined slowly and by only approximately one third (equivalent to ~ 450 µmol/L) after 24 h, following a 6–8 h lag phase (Fig. 3e). A minimal increase in TMA concentrations were observed across 0–8 h and then increased moderately up to 24 h, reaching a concentration similar to the loss of γ-BB (Fig. 3e). l-carnitine concentrations did not change over the 24 h period. Similar to that observed with choline, the background choline in γ-BB-supplemented fermentations declined after a lag of around 8–10 h (Supplementary Fig. 4). These observations are consistent with TMA being produced directly from γ-BB (albeit rather slowly) and not via l-carnitine as an intermediate.

The concentrations of TMA from l-carnitine and γ-BB inoculated fermentations were also measured at 48 h in a single experiment with two replicates per treatment (Supplementary Fig. 5). In the l-carnitine supplemented fermentations, production of TMA increased further between 24 and 48 h to a final concentration of 1541 µmol/L, which corresponded to 93 mol% of the starting concentration of available l-carnitine (1658 µmol/L, Table 1). In the γ-BB-supplemented fermentations, the concentration of TMA reached 1311 µmol/L at 28 h, accounting for 85 mol% of available γ-BB (1536 µmol/L, Table 1). These observations demonstrate that the rate of production of TMA from choline > carnitine > γ-BB, which is reflected in the concentrations of TMA at 24 h, but that given longer incubation (e.g., 48 h) there is almost complete transformation of all three substrates.

### No evidence of inhibition of choline metabolism to TMA by dimethyl-1-butanol (DMB)

**Fig. 4:**
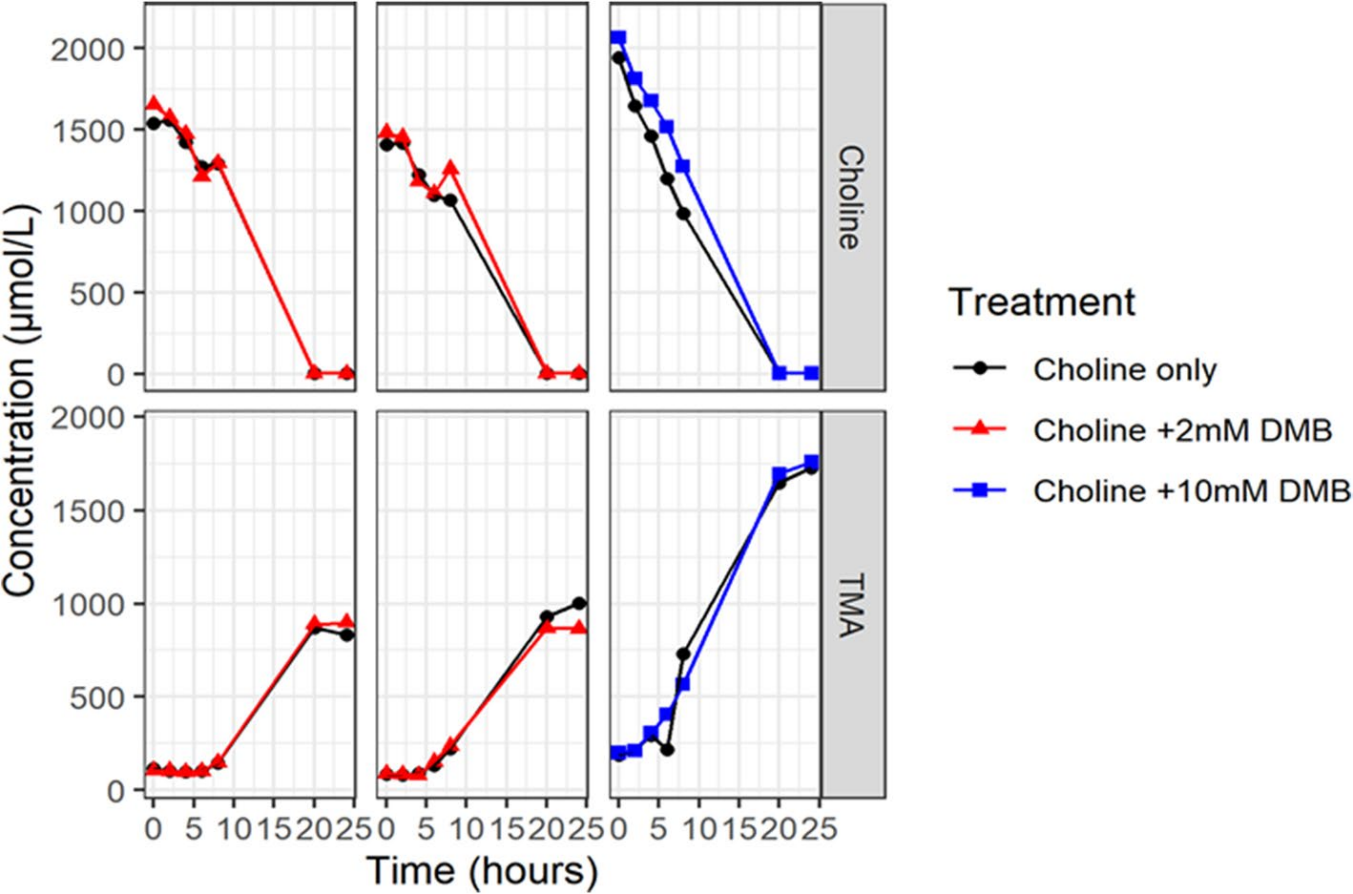
The effects of DMB on TMA production from choline in three independent experiments. The graph is stratified by experiment to enable a direct comparison between paired fermentations with and without DMB added. There was no effect of adding DMB on TMA production or choline concentration at any stage over the time period tested

DMB has been reported to be an effective inhibitor of TMA production from choline; it has been tested on the basis that it is a structural analogue of choline and this is the basis of its mode of action, i.e., as an inhibitor of microbial choline TMA-lyases (cutC/cutD). Figure 4 illustrates the effects of DMB on choline metabolism. Neither the 2 mM nor the 10 mM DMB concentrations influenced choline metabolism and TMA production at any timepoint compared to choline alone. These data show that even high concentrations of DMB are not effective at inhibiting production of TMA from choline in this faecal-inoculated model of the human colon.

### pH control is required TMA production in batch fermentation of human colon models

**Fig. 5:**
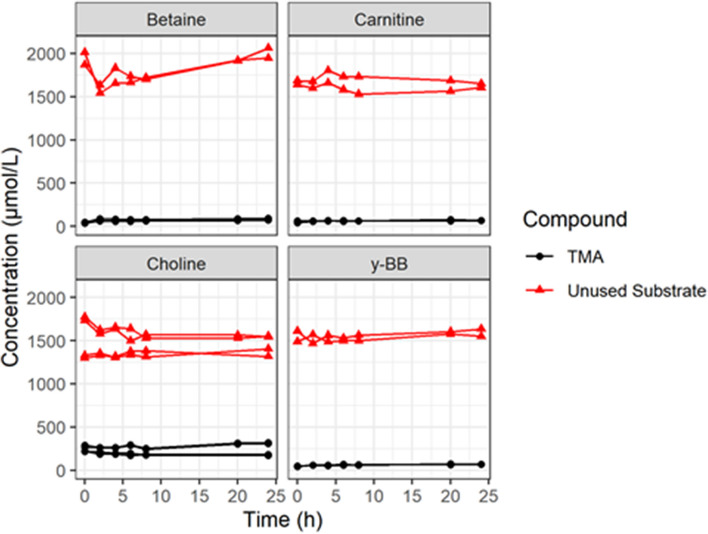
The fermentation of TMA substrates under anaerobic conditions without pH control. There was no fermentation of any of the substrate and no TMA production in anaerobic conditions without pH control, although only a few experiments were carried out (*n* = 3 for choline and 2 for betaine, l-carnitine and γ-BB)

We also examined the potential conversion of the various TMA precursors using stirred vials in anaerobic cabinets in which the fermentations proceed without pH control. Under this condition neither choline, l-carnitine, betaine or γ-BB declined over time and there was no increase in TMA, clearly showing that none of the substrates were metabolised to produce TMA (Fig. 5). This contrasts with reports for cultures of single-strain bacteria and human faecal bacterial cell isolates where in incubations without pH control, these substrates were reported to be metabolised to TMA [22].

## Discussion

The overall aim of the research reported here was to investigate the metabolism of choline, l-carnitine, betaine and γ-BB to TMA in an *in-vitro* batch fermentation model, and in doing so determine how closely the model replicates the production of TMA that has been previously reported in humans. The main findings were that (i) TMA was produced from all three dietary precursors, as well as from the metabolic intermediate γ-BB, (ii) that the relative rates of production of TMA from the substrates was choline > carnitine > γ-BB > betaine, (iii) that all the previously described metabolic routes to TMA production reported in humans were replicated in this *in-vitro* pH-controlled batch colon model and (iv) that no metabolism to TMA occurred from any substrate when fermentations were performed in the absence of pH control.

Unlike in mice, we show that betaine in humans is not formed as an intermediate of choline to TMA metabolism. Instead, almost all choline is metabolised via the direct choline to TMA route, potentially involving the choline TMA-lyases, suggesting that it is the predominant route. There was minimal TMA production in the betaine inoculations and very little betaine disappearance, indicating that in humans the contribution of betaine to TMA production is minimal if at all. Interestingly, background betaine also declined in the inoculations where choline, and l-carnitine were used as substrates. This may suggest that betaine metabolism requires bacteria or enzymes involved in the metabolism of choline, l-carnitine, and γ-BB, i.e., that are only induced in response to available substrates.

In line with others, we demonstrate that the metabolism of l-carnitine to TMA involves the formation of γ-BB as an intermediate and that the Rieske-type carnitine oxygenase/reductase pathways are not involved in this metabolism by human colonic microbiota [22].

We further demonstrate that while the metabolism of choline to TMA is fast, the production of TMA from l-carnitine is much slower. We provide evidence that this is due to the requirement for γ-BB to be formed as an intermediate. More specifically, we have shown that the rate of conversion of γ-BB to TMA is very slow (Fig. 3e), and this is consistent with our observation of significant accumulation of γ-BB in carnitine supplemented fermentations (Fig. 3d), i.e., the very slow rate of conversion of γ-BB to TMA is rate-limiting in carnitine conversion. Nevertheless, if given sufficient time for complete conversion (24 h for choline and 48 h for l-carnitine), similar levels of TMA were achieved. In a review by Fennema et al. it was proposed that betaine may be formed as an intermediate of l-carnitine by l-carnitine dehydrogenase and metabolised further by betaine reductase to form TMA [29], but this was not observed in the model used here.

After inoculation, the concentration of choline started to decline at 6 h, and in most cases it had completely disappeared within 20 h. Increases in TMA concentrations started after 6 h and coincided with the timepoint at which choline concentrations started to decline, strongly suggesting that TMA was directly synthesised from choline. Betaine has been proposed as a possible intermediate in the metabolism of choline to TMA in mice [19–21, 29, 30]. However, at no time-point did we detect betaine in choline supplemented fermentations. Since the rate of production of TMA from betaine was slow relative to the rate of production of TMA from choline (Fig. 3b, c, respectively), these data are in keeping with the notion that all the TMA produced in choline supplemented fermentations is a result of direct conversion via a single enzyme-catalysed step, i.e., via the choline TMA-lyase (CutC/CutD).

As in all experiments where choline was not supplemented as a substrate, background choline declined significantly, we can postulate that some of the TMA may have originated from choline and some from the metabolism of betaine via the glycine betaine transmethylase and then to TMA by decarboxylation. However, we did not measure the concentration of dimethylglycine in this study. Further studies using isotopically labelled betaine would be required to ascertain this. As the expression of CutC/D enzymes has been demonstrated in 100% of all people examined, while between 6 and 21% of people are estimated to lack the betaine metabolising *grdH* enzymes, it is possible faecal samples from participants involved in our study lacked the *grdH* enzymes [31, 32].

Interestingly, although there were high intra-class correlation in TMA production between replicates within the same experiment there were no such correlations with respect to different stool samples from the same donors. This indicates that the capacity to produce TMA in faecal samples collected from the same donor on different days was variable. This is consistent with previous reports that have shown there is high intra-individual variation in plasma TMAO levels over a 1–2 year period, although the source of these variations is not clear and warrants further investigation [33–35]. Nevertheless, future human studies investigating the impact of interventions aimed at reducing TMA production will need to take these variabilities into account by multiple sampling over a period of time and using stable isotopes where necessary.

We sought to replicate previous studies to determine whether DMB could also inhibit the metabolism of choline to TMA in the *in-vitro* batch fermentation model. We were unable to show inhibition of choline metabolism to TMA by DMB at 2 and 10 mM. This shows that investigating TMA precursors in the context of complex bacterial mixtures is crucial as only a fraction of bacterial strains may be inhibited by DMB [21]. Wang et al. further demonstrated that DMB administered orally, but not subcutaneously, was effective at reducing TMAO production from choline indicating that this is a gut microbial dependent inhibition [21]. These discrepancies could be due to differences in the microbiota composition between humans and animals.

In the fermentations with l-carnitine we demonstrate the disappearance of l-carnitine from 6 h after the start of the fermentations, which did not correspond with the observed increase in TMA concentration. However, the disappearance of l-carnitine corresponded with the appearance of γ-BB at 8 h which peaked and plateaued at 12 h and slowly disappeared at 20–24 h, corresponding with TMA production. These observations confirm previous findings in animal and human studies showing that the formation of γ-BB as an intermediate of l-carnitine was obligatory for the formation of TMA. We further confirmed the direct metabolism of γ-BB to TMA by carrying out fermentations in the presence of γ-BB substrate, and we show metabolic dynamics similar to choline where the disappearance of γ-BB corresponds with the appearance of TMA, albeit at a far lower rate in comparison to choline at 24 h. In contrast to the proposed pathway of TMA formation from γ-BB via l-carnitine, where γ-BB may be metabolised to l-carnitine by γ-BB hydroxylase, which is subsequently metabolised to TMA by the Rieske-type carnitine reductase/oxidase, there was no significant formation of TMA production at 8 and 12 h when l-carnitine was completely used up [14, 29]. In addition, at no point did we observe an increase in l-carnitine in the γ-BB fermentations. This once again highlights the need to study microbial functions in the context of the complex microbiota. These observations complement studies by Koeth et al. and Rath et al. who demonstrated that only a small proportion of individual’s stool samples expressed l-carnitine oxygenase CntA/B and that their function is dependent on the availability of molecular oxygen [12, 22].

**Fig. 6:**
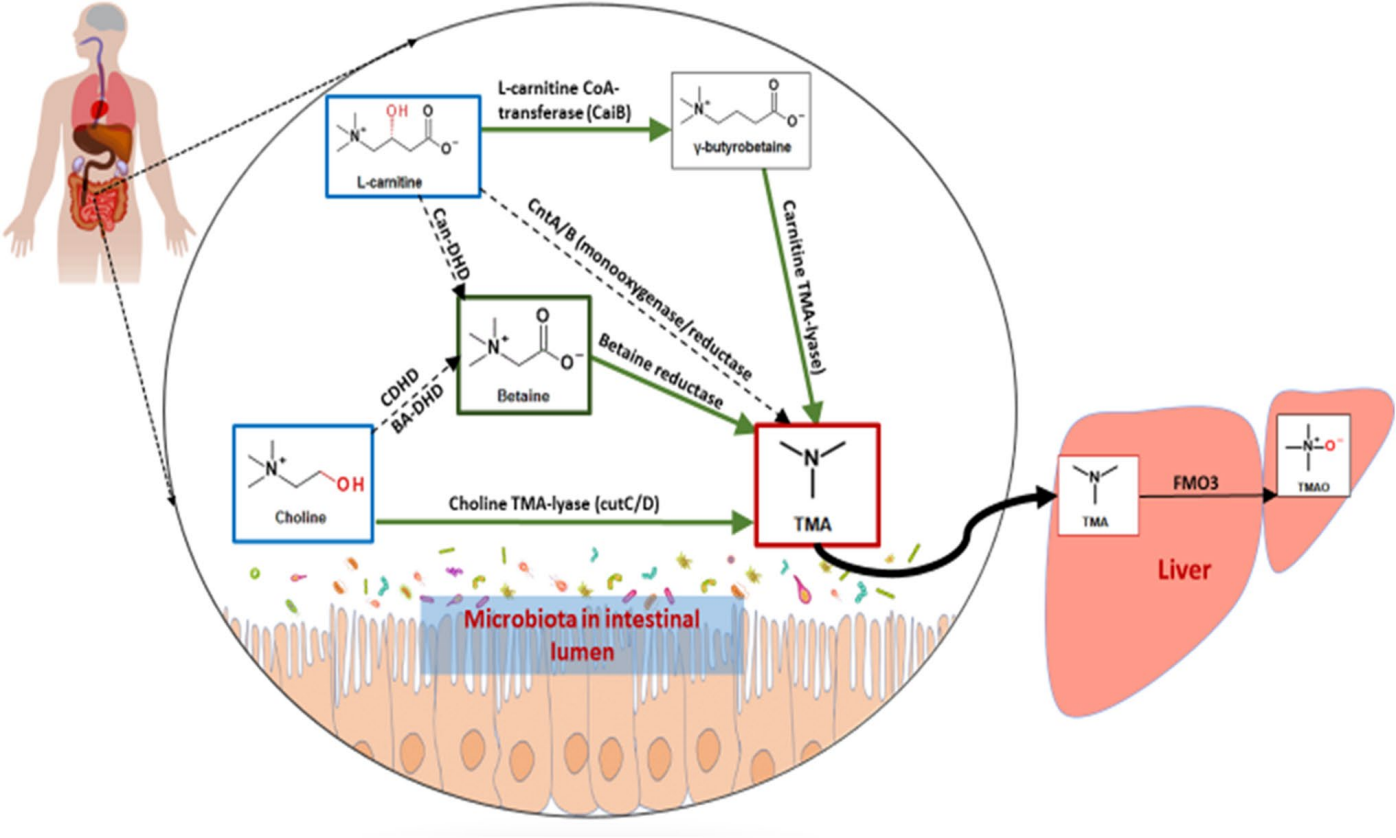
Pathways for the metabolism of choline, betaine, l-carnitine and γ-BB by human gut microbiota. This is based on data reported here and previously by others [5, 10, 12, 19–22, 32]. In humans, choline is metabolised to TMA via the choline TMA-lyase pathway, betaine is not formed as an intermediate of choline to produce TMA via the choline dehydrogenase (CHDH)/betaine aldehyde dehydrogenase (BADH) > betaine reductase pathway, nor is it formed as an intermediate of l-carnitine via the l-carnitine dehydrogenase pathway**.** There is no direct conversion of l-carnitine to TMA via the Rieske-type C l-carnitine oxygenase/reductase pathway, instead l-carnitine is first converted to γ-BB by γ-butyrobetainyl-CoA:carnitine CoA transferases which is then converted to TMA by the l-carnitine TMA lyases. It is possible that betaine may be converted to dimethylglycine by glycine betaine transmethylase and then to TMA by decarboxylation although the evidence for this is weak. Dashed black lines are pathways shown not to be functional in this model; solid green lines indicate pathways we have demonstrated to be important for TMA production in the *in-vitro* human colonic fermentation model

The fact that there was no direct conversion of l-carnitine to TMA demonstrates that the model used here was strictly anaerobic. Our observations regarding TMA production from l-carnitine and choline were in close agreement with previous reports from studies carried out in humans and serve to validate the utility of this model for investigating mechanisms of TMA production in the human colon. Based on the data obtained using this model, we have provided a schematic that shows which metabolic pathways are important for TMA metabolism in humans, and which are not (Fig. 6). We further show that the results generated using the *in-vitro* colon model could not be replicated using the non-pH controlled anaerobic fermentations where none of the substrates declined over time and there was no increase in TMA. This is in contrast to other studies that have used anaerobic fermentations without pH control and have demonstrated choline and l-carnitine metabolism to TMA [15, 16, 18, 21]. In the absence of complex microbiota mixtures, pure cultures may indeed be able to metabolise choline to TMA without pH control, although where a mixture of microbiota is present, other bacteria may outcompete TMA-producing bacteria. Thus, anaerobic fermentations without pH control are not suitable for investigating the mechanisms involved in the production of TMA from its dietary precursors when using multi-species bacterial mixtures, including human faecal samples.

## Conclusion

Using the pH controlled *in-vitro* batch fermentation human colon model, we show that in humans, the choline TMA-lyase pathway is the major pathway for the production of TMA from choline. Unlike in mice, betaine is not formed as an intermediate of choline for the production of TMA, nor is betaine produced as an intermediate of l-carnitine to produce TMA, although very small quantities of TMA may be produced from betaine. Complementing previously reported data from human and animal studies we show that γ-BB is an intermediate for the metabolism of l-carnitine to TMA. We could not show the direct metabolism of l-carnitine to TMA via the Rieske-type carnitine oxygenase/reductase pathway. While strain-specific models are important for identifying bacterial species and compartments for the production of TMA from its dietary precursors, they must always be accompanied by models which include complex microbiota mixtures similar to those found in the human gut and under pH regulation. This model offers an invaluable tool for increasing our understanding of the metabolic pathways of TMA production including identifying further microorganisms that are involved in TMA production and metabolism, such as Archaea. We envisage that this model will also be useful for studies aiming to identify potential dietary or pharmacological inhibitors of TMA production in the human gut.

### Strengths and limitations

One strength of this study is that we were able to use a validated human colon model to investigate the metabolism of TMA precursors separately and investigate their microbial dependent degradation in the presence of complex microbiota species and independent of host enzymes that may also degrade some of these TMA precursors. Another is that the results presented here are consistent with those reported previously from *in-vivo* studies using human participants and animals, with the exception of betaine, which we show is not an intermediate in the metabolism of choline and l-carnitine. A weakness of this research is that the colon model only reflects conditions in the colon, and our observations may not reflect transformations that potentially occur in the upper gut, particularly the ileum, where the structure of the bacterial communities differ. However, the consistency of our observations with those from animal and human intervention studies suggests that it is an excellent model for studying TMA production in the human colon.

## Supplementary Information

Below is the link to the electronic supplementary material.

Supplementary file1 (DOCX 633 kb)
