# Supplementary material for: The use of an in-vitro batch fermentation (human colon) model for investigating mechanisms of TMA production from choline, l-carnitine and related precursors by the human gut microbiota
Source: Eur J Nutr. 2021 May 2;60(7):3987–99. doi: 10.1007/s00394-021-02572-6 (PMC8437865; doi:10.1007/s00394-021-02572-6)
Supplement: Supplementary file 1 — Supplementary file1 (DOCX 633 kb) [file 394_2021_2572_MOESM1_ESM.docx]

**Supplementary Materials**

**The use of an *in-vitro* batch fermentation (human colon) model for investigating mechanisms of TMA production from choline, l-carnitine and related precursors by the human gut microbiota**

Priscilla Day-Walsh^1^, Emad Shehata^1, 2^, Shikha Saha^1^, George M Savva^1^, Barbora Nemeckova^1^, Jasmine Speranza^1^, Lee Kellingray^1^, Arjan Narbad^1^, *Paul A Kroon^1^

^1^Quadram Institute Bioscience, Norwich Research Park, Norwich, NR4 7UQ, UK

^2^Chemistry of Flavour and Aroma Dept, National Research Centre, 33 El Buhouth St., Dokki, 12622 Giza, Egypt

*Corresponding author: paul.kroon@quadram.ac.uk (PAK).

**Supplementary Table I: Estimates of average TMA produced in 24 h for samples with no added substrate (control), and for each treatment compared to control and each substrate.** P-values correspond to the hypothesis that the average TMA produced by each substrate (compared to the control) is zero and differences between substrates.

| **Contrast** | **Mean difference between substrates** | | **Standard error** | **95% confidence interval** | **p-value** |
| --- | --- | --- | --- | --- | --- |
| Betaine - Control | | 133.9 | 48.7 | 34.5 to 233 | 0.0099 |
| Carnitine - Control | | 307.1 | 93.3 | 116.8 to 497 | 0.0025 |
| Choline - Control | | 966.7 | 133.8 | 693.8 to 1240 | <.0001 |
| γ-BB – Control | | 395.9 | 150.5 | 88.9 to 703 | 0.0132 |
| Carnitine - Betaine | | 173.2 | 118.7 | -69 to 415 | 0.1547 |
| Choline - Betaine | | 832.8 | 143.9 | 539.3 to 1126 | <.0001 |
| γ-BB - Betaine | | 262.0 | 160.0 | -64.4 to 588 | 0.1117 |
| Choline - Carnitine | | 659.7 | 140.8 | 372.4 to 947 | 0.0001 |
| Carnitine - γ-BB | | 88.8 | 135.7 | -188 to 366 | 0.5178 |
| Choline - γ-BB | | 570.9 | 131.2 | 303 to 838.5 | 0.0001 |


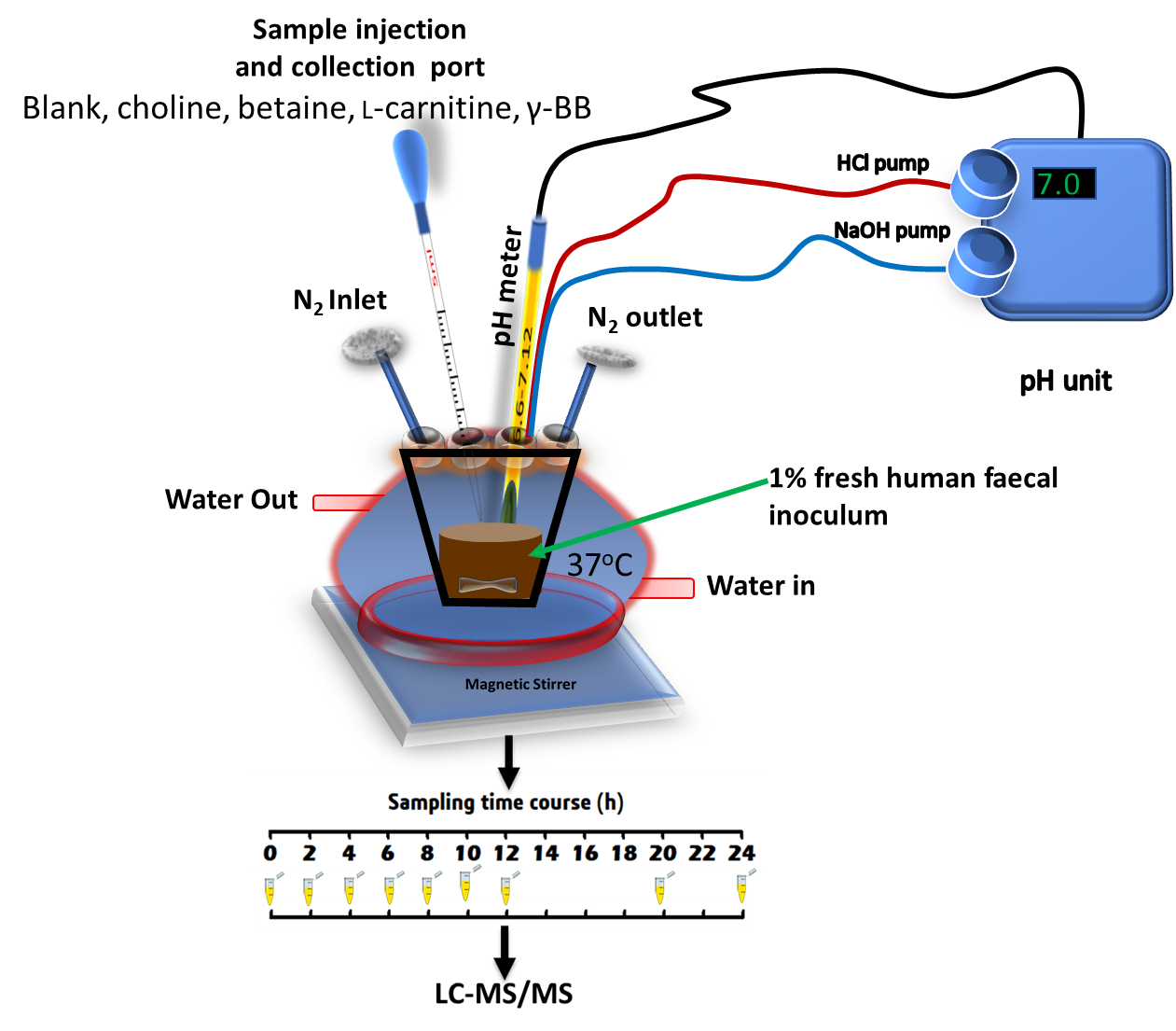


**Supplementary Figure 1: Schematic of the** **human *in-vitro* colon model set up with the total reaction volume of 110ml and gas container volume of 300ml.**


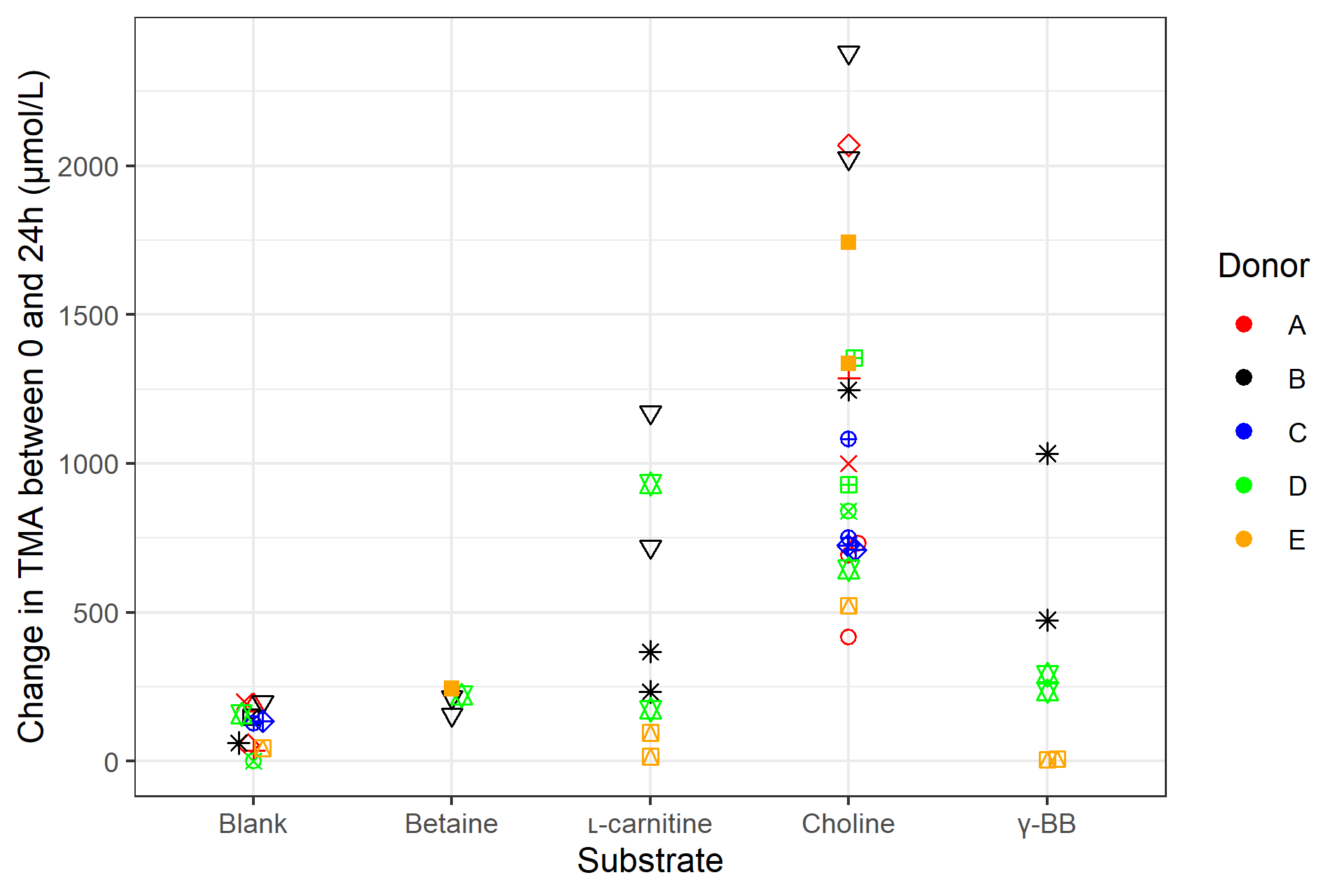


**Supplementary Figure 2: Change in TMA over 24 h following each exposure.** Colour corresponds to donor; shape corresponds to experiment. While there is a high intra-class correlation between replicates within the same experiment (same shape of marker), there is no intra-class correlation with respect to different samples from the same donors.


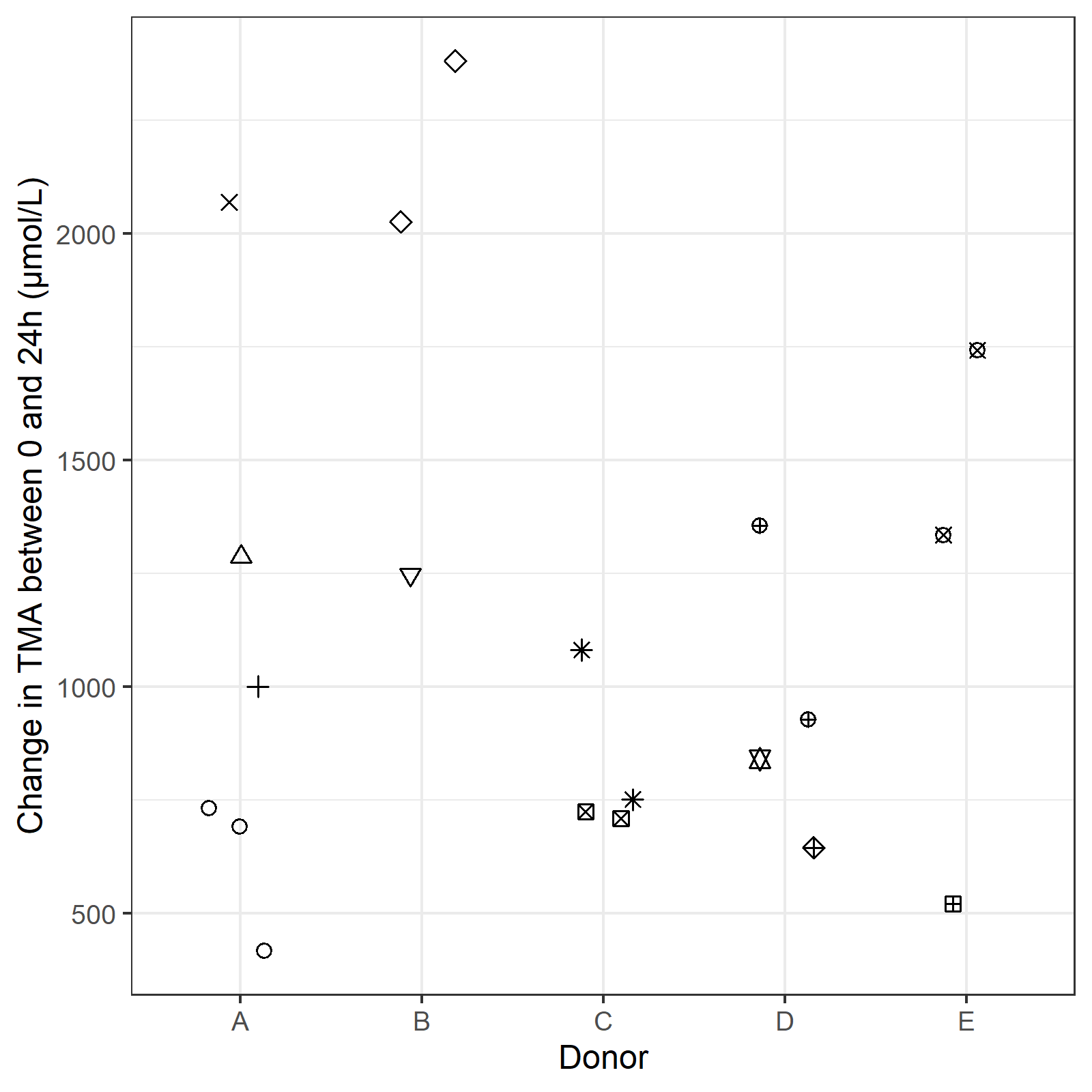


**Supplementary Figure 3:** **Change in TMA between 0 and 24 h with choline as substrate, stratified by donor. Repeats from the same experiment are labelled with the same shape.** While there is no obvious difference between donors, there is a strong correlation between repeats from the same experiment (intra-class correlation within experiments = 0.94, ICC between samples from the same donor = 0).


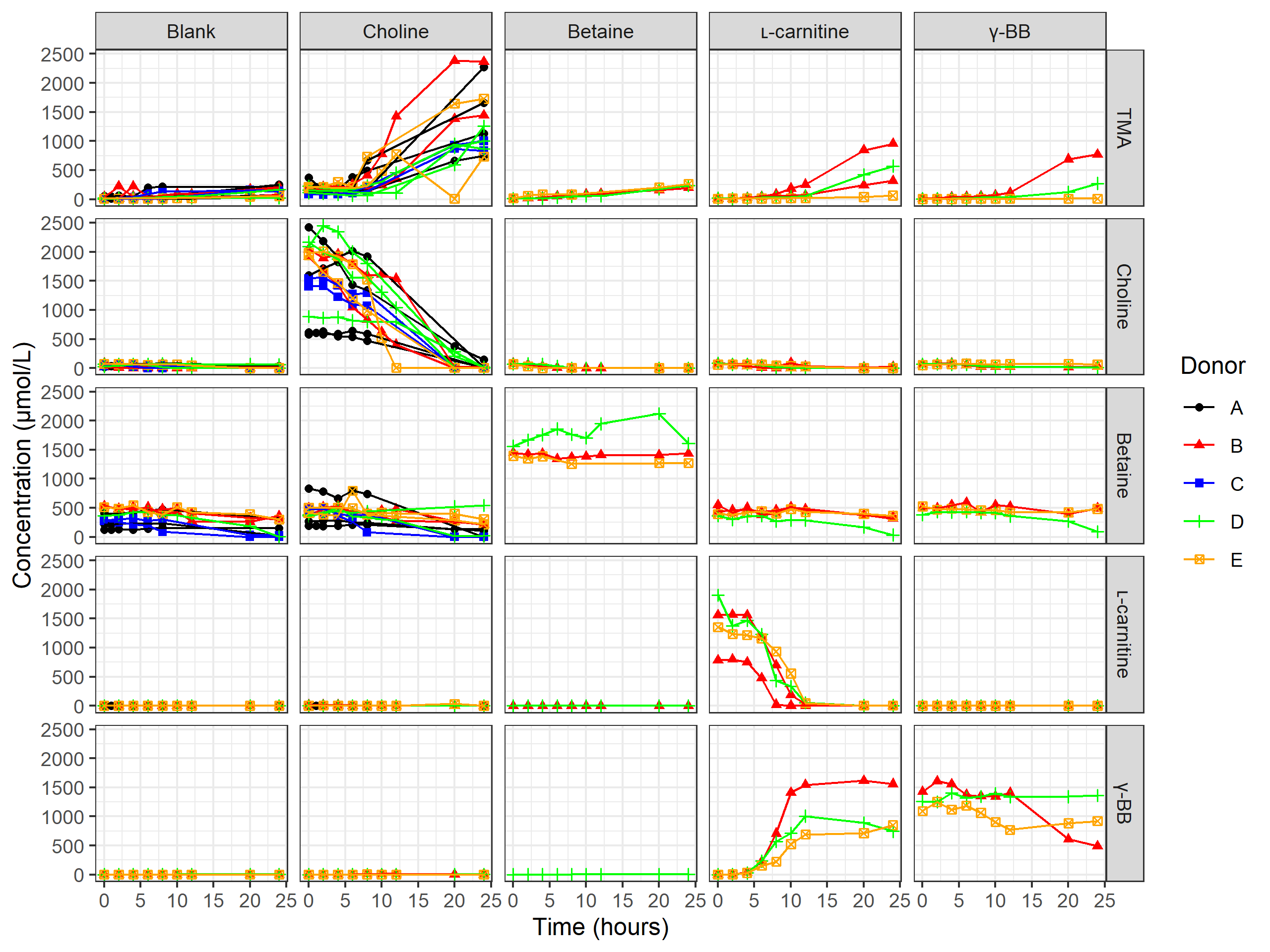


**Supplementary Figure 4: Individual trajectories of each metabolite concentration following addition of each substrate.**


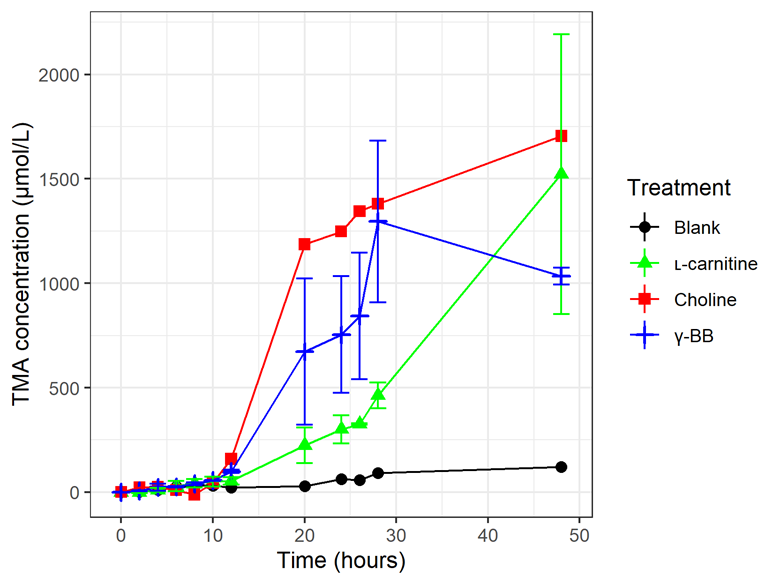


**Supplementary Figure 5:** **TMA production in which fermentations were incubated for 48 hours.** As expected, the conversion of choline to TMA was extensive by 24 h, as was TMA from γ-BB. In contrast, production of TMA from L-carnitine was modest at 24 h but substantial and similar to that of choline and γ-BB after 48 h.
